# Critical Factors Governing the Difference in Antizyme-Binding Affinities between Human Ornithine Decarboxylase and Antizyme Inhibitor

**DOI:** 10.1371/journal.pone.0019253

**Published:** 2011-04-28

**Authors:** Yen-Chin Liu, Yi-Liang Liu, Jia-Yang Su, Guang-Yaw Liu, Hui-Chih Hung

**Affiliations:** 1 Department of Life Sciences and Institute of Genomics and Bioinformatics, National Chung-Hsing University, Taichung, Taiwan; 2 Division of Allergy, Immunology and Rheumatology and Institute of Immunology, Chung-Shan Medical University and Hospital, Taichung, Taiwan; Weizmann Institute of Science, Israel

## Abstract

Both ornithine decarboxylase (ODC) and its regulatory protein, antizyme inhibitor (AZI), can bind with antizyme (AZ), but the latter has a higher AZ-binding affinity. The results of this study clearly identify the critical amino acid residues governing the difference in AZ-binding affinities between human ODC and AZI. Inhibition experiments using a series of ODC mutants suggested that residues 125 and 140 may be the key residues responsible for the differential AZ-binding affinities. The ODC_N125K/M140K double mutant demonstrated a significant inhibition by AZ, and the IC_50_ value of this mutant was 0.08 µM, three-fold smaller than that of ODC_WT. Furthermore, the activity of the AZ-inhibited ODC_N125K/M140K enzyme was hardly rescued by AZI. The dissociation constant (*K*_d_) of the [ODC_N125K/M140K]-AZ heterodimer was approximately 0.02 µM, which is smaller than that of WT_ODC by approximately 10-fold and is very close to the *K*_d_ value of AZI_WT, suggesting that ODC_N125K/M140K has an AZ-binding affinity higher than that of ODC_WT and similar to that of AZI. The efficiency of the AZI_K125N/K140M double mutant in the rescue of AZ-inhibited ODC enzyme activity was less than that of AZI_WT. The *K*_d_ value of [AZI_K125N/K140M]-AZ was 0.18 µM, nine-fold larger than that of AZI_WT and close to the *K*_d_ value of ODC_WT, suggesting that AZI_K125N/K140M has an AZ-binding affinity lower than that of AZI_WT and similar to that of ODC. These data support the hypothesis that the differences in residues 125 and 140 in ODC and AZI are responsible for the differential AZ-binding affinities.

## Introduction

Ornithine decarboxylase (ODC, EC 4.1.1.17) is the first and rate-limiting enzyme in polyamine biosynthesis [1], [2], which is essential for cell growth and differentiation in eukaryotes [3]–[6]. This enzyme catalyzes the pyridoxal 5-phosphate (PLP)-dependent decarboxylation of ornithine to putrescine [1], [2], [5]. ODC and polyamines participate in many important biological processes, including cell proliferation, differentiation, embryonic development, cell cycle, and apoptosis [3], [6]–[12]. The *in vivo* regulation of ODC is very important for the control of cell proliferation [6]. High levels of ODC and polyamines are associated with several human diseases and diverse cancers [6], [9], [13]–[20], and the enzyme activity is related to the beginning and successive development of neoplastic diseases [9], [18]–[20]. Therefore, ODC has been recognized as an oncogenic enzyme, and the study of enzyme inhibitors of ODC may be helpful in the development of therapeutic drugs for the treatment of many cancers [6], [21].

The *in vivo* regulation of ODC is unique [22]. The regulatory protein antizyme (AZ), the expression of which is induced by increased polyamine concentrations, takes charge of ODC inhibition and degradation [23], [24]. ODC undergoes ubiquitin-independent proteasomal degradation by directly interacting with AZ [25]–[27]. The binding of AZ to ODC promotes the dissociation of the ODC dimer. The AZ monomer binds to the ODC dimer to form an inactive ODC-AZ heterodimer that is targeted for degradation by the 26S proteasome [1], [24], [28]–[32]. There is a feedback mechanism for the control of ODC levels. When the level of polyamines is elevated, antizymes are overexpressed to inhibit ODC enzyme activity and to promote the proteolytic degradation of ODC [24], [27], [28]. Thus, AZ acts as a negative regulator of polyamine metabolism by suppressing ODC enzyme activity and polyamine transport to restrict polyamine concentrations [1], [24], [28], [33]. Because high ODC activity is associated with the majority of human malignancies [20], AZ is considered to function as a tumor suppressor.

Another regulatory protein involved in the regulation of ODC is antizyme inhibitor (AZI, [34]) AZI is homologous to ODC but lacks decarboxylase activity [29]. AZI binds to AZ with a higher affinity than does ODC and thus rescues ODC from the ODC-AZ complex to recover ODC enzyme activity [29], [35], [36]. Unlike ODC, both the AZI and AZ proteins undergo ubiquitin-dependent degradation within several minutes to one hour [28], [37]. Furthermore, the binding of AZ to AZI suppresses the ubiquitination of AZI, thus inhibiting its degradation [37], [38]. In contrast to AZ, AZI is a positive regulator of ODC that inactivates all members of the AZ family [39], restores ODC activity [29], [36] and prevents the proteolytic degradation of ODC. Thus, AZI may be oncogenic and may play a role in tumor progression [34]. Overexpression of AZI has been demonstrated to enhance cell proliferation and stimulate cell transformation [34], [40], [41]. Moreover, down-regulation of AZI inhibits cell proliferation and decreases ODC activity through the up-regulation of AZ function [42]. These results reveal that AZI is a positive modulator for cell proliferation and tumorigenesis.

**Figure 1:**
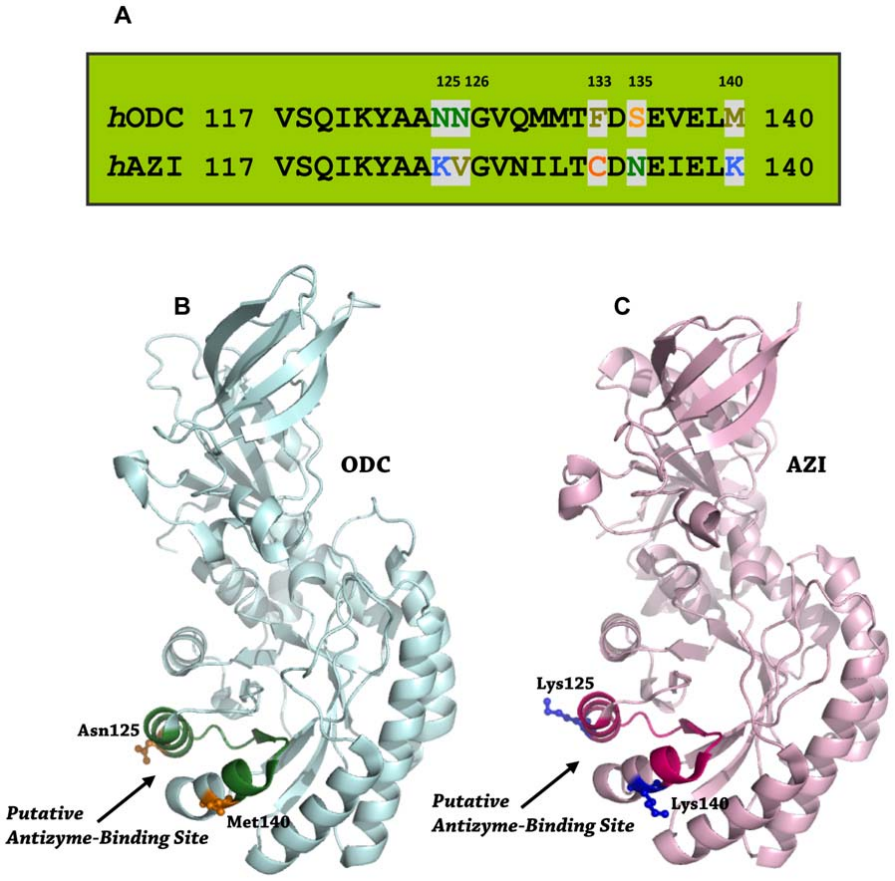
Sequence alignment and structures of ODC and AZI. (**A**) Pairwise sequence alignment between ODC and AZI in the putative AZ-binding element. (**B**) Structure of human ODC monomer (PDB code: 1D7K). (**C**) Structure of mouse AZI monomer (PDB code: 3BTN). The putative AZ-binding site in ODC is colored in deep green and that in AZI is colored in hot pink. This figure was generated with PyMOL (DeLano Scientific LLC, San Carlos, CA, USA).

ODC and AZI are homologous proteins with high sequence identity and structure similarity. ODC is a homodimer containing 461 amino acid residues in each monomer with a molecular weight of 106 kDa [43]. ODC activity requires dimer formation because the active site in each monomer is formed by the interface between the N-terminus of one monomer and the C-terminus of the other subunit [43]–[47]. AZI is a monomer under physiological conditions [48]; it contains 448 amino acid residues and has a molecular weight of 50 kDa. AZI binds more tightly to AZ than does ODC [29], [37]. A structural study of human ODC and mouse AZI has suggested that the region from residue 117 to residue 140 may be the putative AZ-binding site [44], [48]. Furthermore, the docking structures of the mouse AZ-ODC and AZ-AZI complexes suggest that ODC and AZI may occupy the same binding site on AZ [49]. In the present work, we identified the critical amino acid residues governing the difference in AZ-binding affinity between ODC and AZI. Sequence alignments of human ODC and AZI in the putative AZ-binding site, between amino acids 117 and 140, demonstrated that residues 125, 126, 133, 135 and 140 are not conserved between ODC and AZI (Figure 1A). In this study, site-directed mutagenesis was used to generate a series of mutants of ODC and AZI. According to the size-distribution analysis of these ODC and AZI mutants, we have demonstrated that residues 125 and 140 are responsible for the differential AZ-binding affinities between ODC and AZI.

## Results and Discussion

The X-ray structure of human ODC suggests that the putative binding site of AZ may be located between amino acids 117 and 140 of ODC [44]. Modeled structures of the ODC-AZ and AZI-AZ complexes also suggest that both ODC and AZI bind to the same binding site on AZ [49]. Although the structures of ODC and AZI are very similar and may have the same AZ-binding site (Figure 1, A and B), AZI binds to AZ more tightly than does ODC [29]. Sequence alignment of amino acids 117 and 140 in human ODC and AZI revealed that all amino acid residues are conserved with the exceptions of residues 125, 126, 133, 135 and 140 (Figure 1A). These non-conserved amino acid residues may be the factors governing the differential AZ-binding affinities between ODC and AZI. To address the question, these amino acid residues in human ODC were individually changed to the residues present in human AZI. Similarly, the corresponding amino acid residue in human AZI was substituted into human ODC.

### Inhibition of wild-type and mutant ODC enzyme activity by AZ

**Figure 2:**
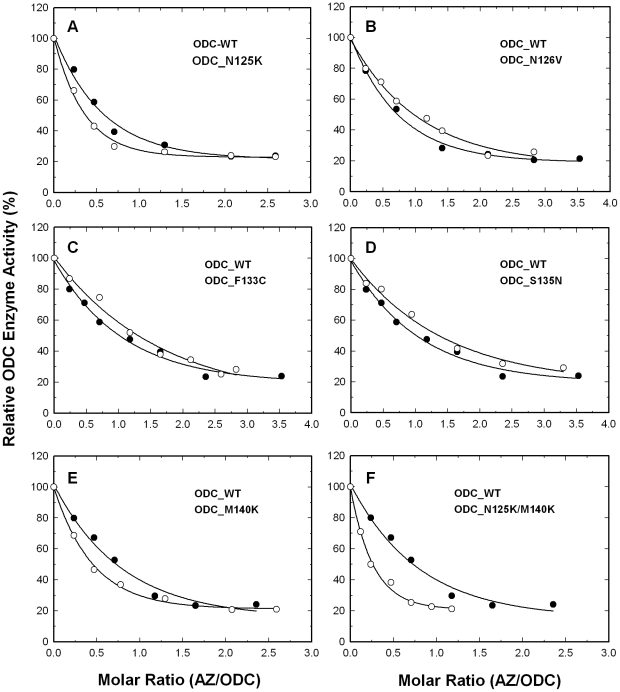
Inhibition of wild-type and mutant ODC enzyme in the presence of AZ. Open circles: ODC_WT; closed circles: mutant ODC enzyme. (**A**) ODC_N125K. (**B**) ODC_N126V. (**C**) ODC_F133C. (D) ODC_S135N. (E) ODC_M140K. (**F**) ODC_N125K/M140K. In the inhibition assay, the enzyme concentration was fixed at 20 µg/mL.

We first mutated these five residues to create a series of single mutants of ODC: ODC_N125K, ODC_N126V, ODC_F133C, ODC_S135N and ODC_M140K. For ODC_WT, the enzyme activity was gradually inhibited by the increased concentrations of AZ (Figure 2, closed circles), and the concentration of AZ required for 50% inhibition of ODC enzyme activity (IC_50,AZ_) was approximately 0.25 µM (Table 1). ODC_N125K was inhibited more obviously than ODC_WT (Figure 2A, open circles), with an IC_50,AZ_ value of 0.1 µM (Table 1). The ODC_N126V, ODC_F133C and ODC_S135N enzymes were less sensitive to AZ inhibition (Figure 2, B, C and D, respectively, open circles); the IC_50,AZ_ values of these mutant enzymes were 0.44, 0.45 and 0.49 µM, respectively, all slightly larger than that of ODC_WT (Table 1). The ODC_M140K enzyme, similar to ODC_N125K, was inhibited to a greater degree than ODC_WT (Figure 2E); the IC_50,AZ_ value of this mutant was 0.13 µM, smaller than that for ODC_WT (Table 1). We also constructed the ODC_N125K/M140K double mutant enzyme. The inhibition of this double mutant by AZ was even greater than that of either single mutant, ODC_N125K or ODC_M140K (Figure 2F, open circles); the IC_50_ value of ODC_N125K/M140K was 0.08 µM, three-fold smaller than that of ODC_WT (Table 1).

**Table 1:** Kinetic parameters and IC_50_ values of the wild-type and mutant ODC enzymes.

ODC	*K*_m,ornithine_ (mM)	*k*_cat_ (s^−1^)	IC_50,AZ_ (µM)*
WT	0.48±0.09	7.4±0.5	0.25±0.07
N125K	0.48±0.08	6.9±0.3	0.10±0.002
N126V	0.42±0.09	5.8±0.3	0.44±0.25
F133C	0.30±0.05	4.8±0.1	0.45±0.13
S135N	0.33±0.17	5.1±0.6	0.49±0.25
M140K	0.44±0.08	6.6±0.3	0.13±0.006
N125K/M140K	0.34±0.03	4.4±0.1	0.08±0.015

*All IC_50_ values were derived from the inhibition curves of ODC in Figure 2.

### AZI rescue for the AZ-inhibited wild-type and mutant ODC enzyme activity

**Figure 3:**
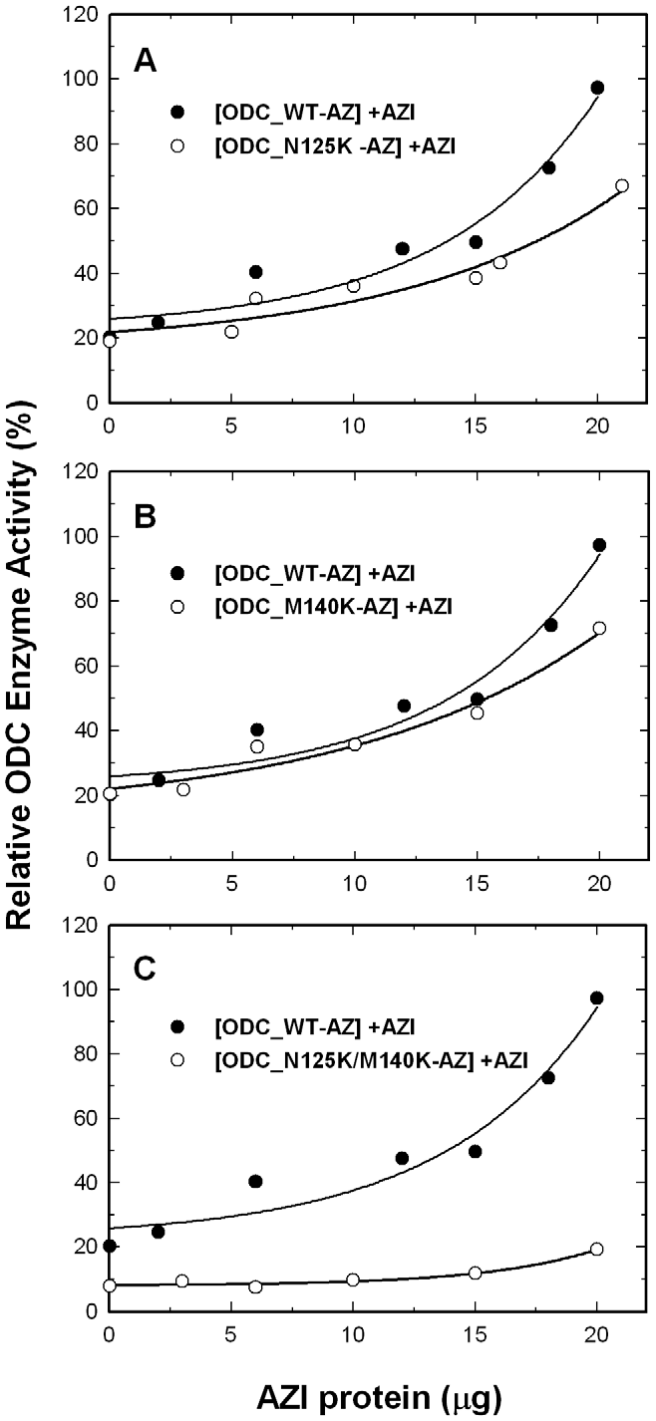
Rescue of AZ-mediated inhibition of the activity of wild-type and mutant ODC by AZI. ODC (20 µg/mL) was preincubated with AZ (34 µg/mL) and was then treated with various concentrations of AZI. Open circles: ODC_WT; closed circles: mutant ODC enzyme. (**A**) ODC_N125K. (**B**) ODC_M140K. (**C**) ODC_N125K/M140K. The molar ratio of AZ monomer versus ODC monomer was fixed at 4.

AZI can rescue ODC monomers from AZ-ODC complexes to restore ODC activity [29]. We examined the effect of AZI on the rescue of the activity of AZ-inhibited wild-type and mutant ODC. The ODC enzyme was first preincubated with AZ, keeping the molar ratio of AZ monomer versus ODC monomer at 4.0. For ODC_WT, with increasing AZI concentrations, the residual enzyme activity increased from 20% to over 90%. For ODC_N125K and ODC_M140K, although the AZ-inhibited ODC activity could be recovered, the residual enzyme activity curves of the two mutants were below the curve of ODC_WT (Figure 3, A and B, respectively, open circles). When the enzyme activity of ODC-AZ was restored by AZI to nearly 100%, the activities of ODC_N125K and ODC_M140K were recovered by approximately 60% to 70% (at 20 µg of AZI). The activity of the AZ-inhibited ODC_N125K/M140K enzyme activity was hardly rescued by AZI; even at high concentrations of AZI, the residual enzyme activity was only 20% (Figure 3C, open circles). The resistance of ODC_N125K/M140K to rescue by AZI may result from the tighter binding of this double mutant to AZ as compared with ODC_WT.

These results for the AZ inhibition and AZI rescue experiments indicate that the replacement of Asn125 and Met140 in ODC with the respective amino acid residues, Lys125 and Lys140 that exist in AZI, results in an ODC enzyme that is more sensitive to AZ inhibition and more resistant to the rescue by AZI. This demonstrates the significance of these two residues in determining the differential AZ-binding affinity between ODC and AZI.

### The effect of wild-type and mutant AZI on the rescue of the activity of AZ-inhibited ODC

**Figure 4:**
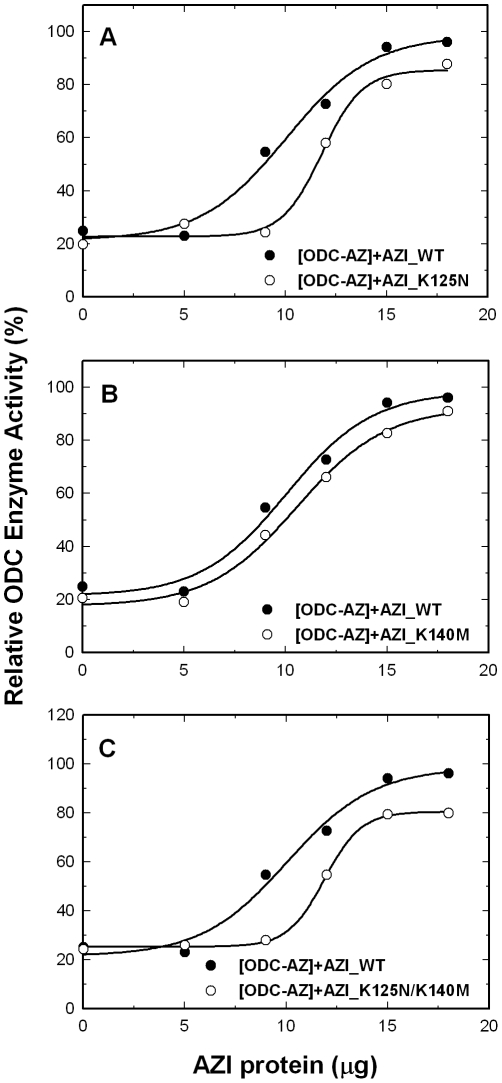
Rescue of AZ-mediated inhibition of the activity of ODC by wild-type and mutant AZI. ODC (20 µg/mL) was preincubated with AZ (30 µg/mL) and then treated with various concentrations of AZI. Open circles: AZI_WT; closed circles: mutant AZI. (**A**) AZI_K125N. (**B**) AZI_ K140M. (**C**) AZI_K125N/K140M. The molar ratio of AZ monomer versus ODC monomer was fixed at 3.5.

We further created the single mutants AZI_K125N and AZI_K140M and the double mutant AZI_K125N/K140M. To optimize the rescue of these mutant AZI proteins, the molar ratio of ODC versus AZ was fixed at 3.5 (Figure 4). For AZI_K125N, although the AZ-inhibited ODC activity could be restored, the residual enzyme activity curve of this mutant was below the curve for AZI_WT (Figure 4A, open circles), indicating that the rescue efficiency of this AZI mutant was less than that of AZI_WT. Using AZI_K140M, the AZ-inhibited ODC activity could also be restored (Figure 4B, open circles). However, the rescue efficiency of this AZI mutant was just slightly less than that of AZI_WT, indicating a minor effect of this replacement on AZI for its rescue efficiency. The efficiency of the double mutant AZI_K125N/K140M to rescue the activity of the AZ-inhibited ODC was very similar to that of AZI_ K125N (Figure 4C, open circles). These data suggest that these mutations in AZI reduced the efficiency of the rescue of the activity of AZ-inhibited ODC.

### Binding affinity of ODC-AZ and AZI-AZ complex

**Figure 5:**
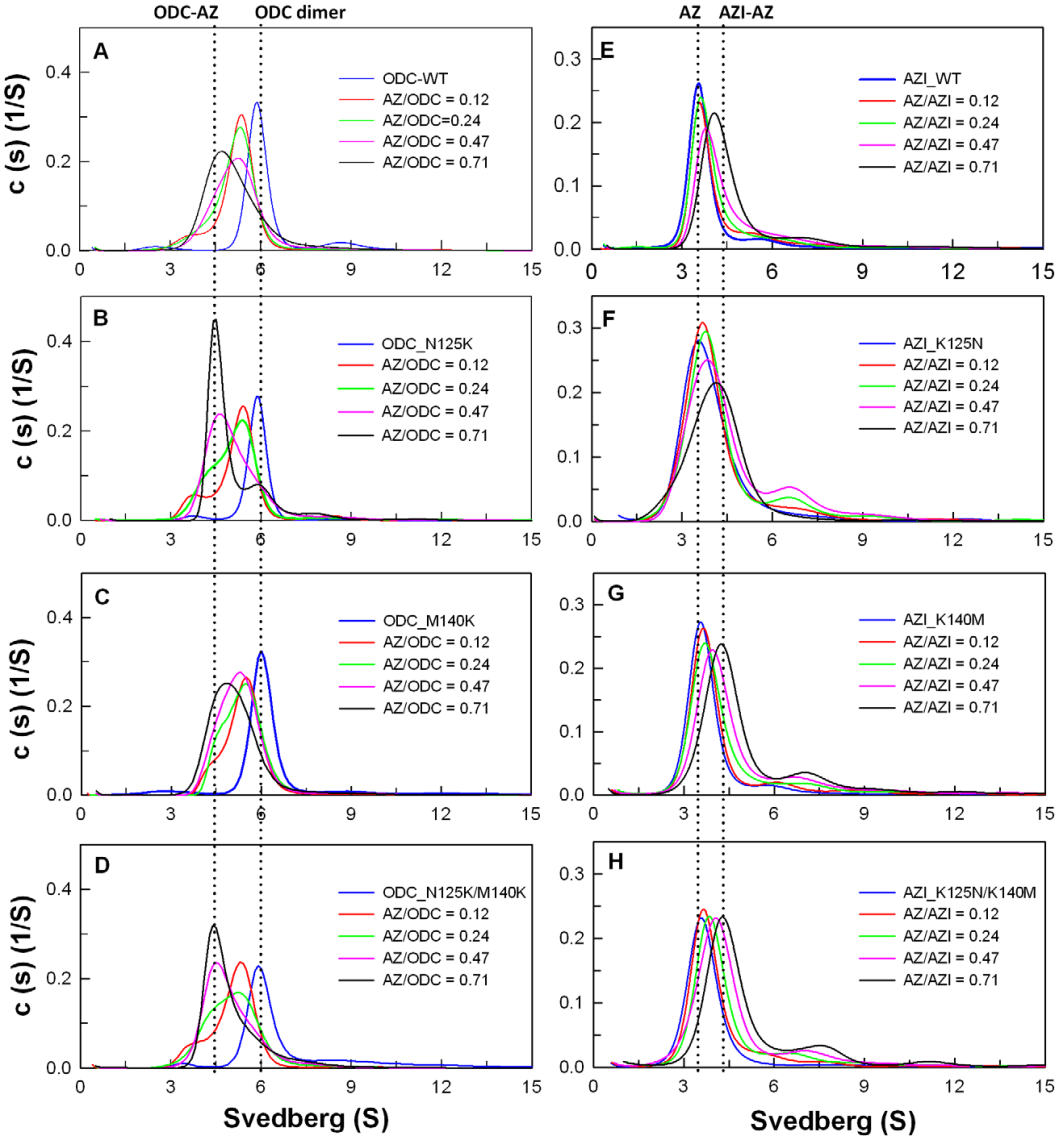
Continuous sedimentation coefficient distribution of human ODC and AZI in the presence of AZ. The concentration of ODC or AZI was fixed at 0.3 mg/mL with four concentrations of AZ at 0.015, 0.03, 0.06, or 0.09 mg/mL (the molar ratios of AZ/ODC were 0.12, 0.24, 0.47 and 0.73). The sedimentation velocity data were globally fitted with SEDPHAT to acquire the *K*_d_ values of the ODC-AZ and AZI-AZ complexes (Table 2). (**A**) ODC_WT. (**B**) ODC_N125K. (**C**) ODC_M140K. (**D**) ODC_N125K/M140K. (**E**) AZI_WT. (**F**) AZI_K125N. (**G**) AZI_ K140M. (**H**) AZI_K125N/K140M.

Size distribution analysis of ODC in the presence of AZ provided information about the formation of ODC-AZ complexes (Figure 5A). AZ binds to ODC, which induces the dissociation of the ODC dimer and results in the formation of an ODC-AZ heterodimer. AZI can also interact with AZ to form an AZI-AZ heterodimer [29]. To evaluate the effect of ODC and AZI mutations on AZ-binding affinity, sedimentation velocity (SV) experiments with various AZ concentrations were used to determine the dissociation constants of the ODC-AZ and AZI-AZ heterodimers (Table 2). Figure 5 shows the size distribution plots of the WT and mutant ODC and AZI proteins. Without AZ, ODC existed as a dimer at an S value of approximately 6. When AZ was present, ODC was dissociated, the dimer peak for ODC shifted to the left, and the ODC-AZ heterodimer was produced at an S value of approximately 4.5 (Figure 5A). The *K*_d_ value of the [ODC_WT]-AZ complex was approximately 0.21 µM. For [ODC_N125K]-AZ and [ODC_M140K]-AZ, the *K*_d_ values were approximately 0.1 µM, two-fold smaller than that of the [ODC_WT]-AZ complex, suggesting that these two ODC mutant enzymes had a higher binding affinities for AZ than did the wild type. The double mutant ODC_N125K/M140K had an AZ-binding affinity much higher than that of ODC_WT. The *K*_d_ value of the [ODC_N125K/M140K]-AZ heterodimer was approximately 0.02 µM, smaller than that of WT_ODC by approximately 10-fold and very close to the *K*_d_ value of AZI_WT, suggesting that ODC_N125K/M140K has an AZ-binding affinity that is higher than that of ODC_WT and similar to that of AZI. Thus, ODC_N125K/M140K was more sensitive to AZ inhibition (Figure 2F) and more resistant to rescue by AZI (Figure 3C).

**Table 2:** Dissociation constants of human ODC-AZ and AZI-AZ complexes.

[ODC-AZ] complex	*K*_d,ODC-AZ_ (µM)	[AZI-AZ] complex	*K*_d,AZI-AZ_ (µM)
ODC_WT	0.21±0.001	AZI_WT	0.02±0.009
ODC_N125K	0.10±0.001	AZI_K125N	0.16±0.009
ODC_M140K	0.14±0.001	AZI_K140M	0.11±0.001
ODC_N125K/M140K	0.02±0.002	AZI_K125N/K140M	0.18±0.01

The dissociation constants (*K*_d_) of ODC-AZ and AZI-AZ were derived from the global fitting of the sedimentation velocity data to the model of A+B↔AB hetero-association in the SEDPHAT program.

AZI was present predominately as a monomer at an S value of approximately 3.6; when AZ was present, the monomeric peak of AZI shifted to right, and the AZI-AZ heterodimer was formed at an S value of approximately 4 to 4.5 (Figure 5E). The *K*_d_ value of the [AZI_WT]-AZ complex was approximately 0.02 µM, 10-fold smaller than that of [ODC_WT]-AZ. For [AZI_K125N]-AZ and [AZI_K140M]-AZ, the *K*_d_ values were approximately 0.16 and 0.11 µM, respectively, five- to eight-fold larger than that of the [AZI_WT]-AZ heterodimer, suggesting that these two AZI mutant enzymes had a lower binding affinities for AZ. The double mutant AZI_K125N/K140M had an AZ-binding affinity similar to that of AZI_K125N, with a *K*_d_ value of 0.18 µM, nine-fold larger than that of AZI_WT and close to the *K*_d_ value of ODC_WT, suggesting that AZI_K125N/K140M had an AZ-binding affinity lower than that of AZI_WT and similar to that of ODC. Thus, the efficiency of AZI_K125N/K140M in the rescue of the ODC enzyme activity was less than that of AZI_WT (Figure 4C).

### Solvent accessibility in the putative AZ-binding site of ODC and AZI

Using the structures of ODC and AZI, the solvent accessibility of the putative AZ-binding site was analyzed (http://www.ebi.ac.uk/msd-srv/prot_int/pistart.html, [50]). Based on the values of the accessible and buried surface areas for the residues 117 to 140, these residues were divided into three groups. For ODC, ten residues are totally exposed to solvent (Tyr122, Ala124, Asn125, Asn126, Gly127, Gln129, Met130, Thr132, Phe133, and Met140), nine of them are partially exposed to solvent (Val117, Ser118, Gln119, Lys121, Asp134, Ser135, Glu136, Val137 and Glu138), and five of them are totally buried in the protein (Ile120, Ala123, Val128, Met131, and Leu139). However, there are some differences in AZI. For AZI, fifteen residues are totally exposed to solvent (Val117, Lys121, Tyr122, Ala124, Lys125, Val126, Gly127, Asn129, Ile130, Thr132, Cys133, Asp134, Glu136, Glu138 and Lys140), four of them are partially exposed to solvent (Ser118, Gln119, Asn135, and Ile137) and five of them are totally buried in the protein (Ile120, Ala123, Val128, Met131, and Leu139). Analysis of these two proteins suggested that the putative AZ-binding site of AZI is more solvent-accessible than that of ODC. Docking structures of mouse AZ-ODC and AZ-AZI suggest that AZ is bound within a large groove of ODC and AZI [49]. Residues 125 and 140 in ODC and AZI are solvent-accessible and are present on the outer part of the large groove (Figure 1, B and C, respectively), suggesting that these two residues may directly participate in the interaction with AZ.

### Differences in the electrostatic surface drive the differential AZ-Binding affinities of ODC and AZI

Our data clearly indicate that residues 125 and 140 play critical roles governing the differential AZ-binding affinities of human ODC and AZI. According to the docking result of AZ-AZI model (49), Lys125 in AZI is located near Glu164 and Glu165 of AZ while in ODC a Ser is present at this position. In addition, residues 125 and 140, which are both Lys in AZI, and Asn and Met, respectively, in human ODC are both acidic residues in trypanosome ODC. This is important as trypanosome ODC does not bind AZ. Thus, Lys125 and Lys140 in the putative AZ-binding site of AZI may have electrostatic effects in the binding of AZ. Substituting Lys for Asn125 and Met140 in ODC (ODC_N125K/M140K) introduces extra charges into the AZ-binding element of ODC making this region more similar to that in AZI, thus making the ODC enzyme to more susceptible to inhibition by AZ and more resistant to rescue by AZI. Because Lys125 and Lys140 are of AZI conserved among different species and because in most ODC enzymes, residue 125 is Asn or Ser and residue 140 is Met, we believe that these two positively charged lysine residues are important for tight binding between AZI and AZ.

In summary, according to the mutagenesis analysis and sequence comparisons, we suggest that electrostatic effects are responsible for the differential binding affinities between AZ and ODC and between AZ and AZI. The results observed for ODC_N125K/M140K and AZI_K125N/K140M support this conclusion. The differences in these two residues between ODC and AZI are responsible for the differential AZ-binding affinities.

## Materials and Methods

### Site-directed mutagenesis of the putative AZ-binding site of ODC and AZI

Mutated human ODC and AZI plasmids were generated by site-directed mutagenesis using the QuikChange™ kit (Stratagene, USA). In the PCR reaction, purified human ODC and AZI DNAs were used as the templates, and the high-fidelity Pfu DNA polymerase and the specific primers with desired codons were used to produce the specific mutated DNA. The lengths of the primers with the preferred mutation site were designed between 25 to 45 bases; this number of bases is required for specific binding to the template DNA. After 16–18 temperature cycles, the mutated plasmids with staggered nicks were made. The wild-type human ODC and AZI templates in the PCR products were cleaved by treating with DpnI. The nicked DNAs with specific mutations were used to transform the XL-1 *E. coli* strain, and the DNA sequences were confirmed by autosequencing.

### Expression and purification of recombinant ODC, AZ and AZI

Human ODC, AZ and AZI genes were sub-cloned into the pQE30 vector (Qiagen, Hilden, Germany) with an N-terminal His6·Tag sequence, which is required for protein purification. The expression vector, containing an ampicillin resistance gene, was transformed into the JM109 strain of *Escherichia coli*, which was then exposed to 1.0 mM isopropyl-1-thio-β-D-galactoside (IPTG) to induce protein expression. The overexpressed His6·Tag proteins were purified using Ni-NTA Sepharose (Sigma). The lysate-Ni-NTA mixture was first washed with buffer containing 10 mM imidazole, 500 mM NaCl and 30 mM Tris-HCl (pH 7.6) to eliminate most of the unwanted proteins. Subsequently, ODC, AZ or AZI was eluted using elution buffer, which contained 250 mM imidazole, 500 mM NaCl, 2 mM β-mercaptoethanol and 30 mM Tris-HCl (pH 7.6). The purified ODC or AZI was buffer-exchanged and concentrated with 30 mM Tris-HCl (pH 7.6) and 2 mM β-mercaptoethanol, and the purified AZ protein was buffer-exchanged and concentrated with 250 mM NaCl, 30 mM Tris-HCl (pH 7.6), and 2 mM β-mercaptoethanol. The protein purity was assessed by sodium dodecyl sulfate-polyacrylamide gel electrophoresis (SDS-PAGE), and the protein concentrations were estimated using the Bradford method [51].

### ODC enzyme reaction

The ODC enzyme activity was determined using the CO_2_-L3K assay kit (DCL, Charlottetown, Canada) at 37°C. The continuous measurement of ODC enzyme activity was combined with the phosphoenolpyruvate carboxylase- and malate dehydrogenase-catalyzed reactions; the protocol is described in our recent paper [29]. The reaction cocktail for the spectrophotometric assay of ODC activity comprised 30 mM Tris-HCl (pH 7.8), 10 mM ornithine, 0.2 mM PLP, and 0.4 mL of CO_2_-L3K assay buffer in a total volume of 0.5 mL. In this coupled assay reaction, 1 mol of CO_2_ was produced concomitant with the oxidation of 1 mol of NADH analog, and the decrease in absorbance at 405 nm corresponding to the oxidation of the NADH analog was continuously traced using a Perkin-Elmer Lambda-25 spectrophotometer. An extinction coefficient of 2410 m ^−1^ was used for the NADH analog in these calculations. Sigma Plot 10.0 (Jandel, San Rafael, CA) was used to carry out all of the calculations. In the ODC inhibition experiment, the concentration of ODC was fixed (20 µg/mL), and the AZ concentration was varied. For the AZI rescue experiment, ODC (20 µg/mL) was first preincubated with AZ (30–34 µg/mL) to inhibit 80% of the enzyme activity, and then the mixture was treated with various concentrations of AZI. The molar ratio of ODC versus AZ was calculated using the molecular weights of monomeric ODC and AZ.

### Size-distribution analysis by analytical ultracentrifugation

A Beckman Optima XL-A analytical ultracentrifuge device was used to perform the sedimentation velocity experiments. Buffer (400 µl) and sample solutions (380 µl) were loaded into the double sector centerpiece separately and established in a Beckman An-50 Ti rotor. A rotor speed of 42,000 rpm was used in the sedimentation velocity experiments. Protein samples were analyzed using UV absorbance at 280 nm in continuous mode with a time interval of 420 s and a step size of 0.002 cm. Numerous scans at different collecting times were fitted to a continuous size distribution model using SEDFIT software [52], [53]. All size distributions were estimated at a confidence level of *p* = 0.95, a best fit average anhydrous frictional ratio (*f/f*_0_), and a resolution *N* of 250 sedimentation coefficients between 0.1 and 15.0 S.

The dissociation constants (*K*_d_) of the ODC-AZ and AZI-AZ complexes were estimated using the results of the sedimentation velocity experiments, which were done using a constant concentration of human ODC or AZI with four different concentrations of AZ. All sedimentation data were globally fitted into the AB hetero-association model using SEDPHAT [54], [55] to obtain the *K*_d_ value of the ODC-AZ or AZI-AZ heterodimer. The partial specific volumes of the proteins, the solvent densities, and the viscosity were calculated using SEDNTERP [56].
